# Can Front-of-Pack Labeling Encourage Food Reformulation? A Cross-Sectional Study on Packaged Bread

**DOI:** 10.3390/foods13223535

**Published:** 2024-11-06

**Authors:** Daniela Martini, Donato Angelino, Massimiliano Tucci, Edoardo La Bruna, Nicoletta Pellegrini, Cristian Del Bo’, Patrizia Riso

**Affiliations:** 1 Department of Food, Environmental and Nutritional Sciences (DeFENS), Università degli Studi di Milano, 20133 Milan, Italy; 2 Department of Bioscience and Technology for Food, Agriculture and Environment, University of Teramo, 64100 Teramo, Italy; 3 Department of Agricultural, Food, Environmental and Animal Sciences, University of Udine, 33100 Udine, Italy

**Keywords:** food labeling, nutritional values, reformulation, nutritional quality

## Abstract

Front-of-pack labeling (FOPL) may represent an important instrument for the food industry in the promotion of food product reformulation. The present cross-sectional study used salt reduction in packaged breads as a case study, aiming to investigate whether two different types of FOPL (i.e., Nutri-Score (NS) and NutrInform battery (NIB)) can capture food reformulation and thus be effective tools for encouraging reformulation. The Nutri-Score and NIB were calculated by consulting the nutritional declarations and ingredient lists of 527 packaged breads currently sold in Italy before and after applying three different theoretical reformulation strategies: (i) a 25% salt decrease from the current median salt content in bread; (ii) a reduction of up to 0.825 g/100 g of salt, corresponding to the sodium benchmark of 330 mg/100 g set by the World Health Organization (WHO); and (iii) the minimum salt reduction needed to improve the NS by one grade. The results show that only ~44% of breads had improved NSs when the sodium was lowered to reach the WHO benchmark or when salt was reduced by 25%, whereas large variability was observed in the minimum salt reduction needed to improve the NS. Regarding the NIB, the battery for salt improved when both strategies of reformulation were applied. FOPL is not always effective in capturing food reformulation in terms of salt reduction, possibly discouraging the efforts of food companies to improve the nutritional quality of foods.

## 1. Introduction

Europe is facing a large debate about front-of-pack labeling (FOPL) due to the intention/decision within the Farm-to-Fork strategy to define a mandatory and harmonized proposal for FOPL to be used on all food items sold in the European Union. This label is intended to represent one of the actions to empower consumers to make healthy food choices [1,2,3]. So far, many countries have developed FOPL proposals in agreement with Art. 35 of the Reg. (UE) n. 1169/2011, which allows Member States to recommend that food business operators use one or more additional forms of expression or presentation for nutrition declaration as long as they are based on sound and scientifically valid consumer research, do not mislead the consumer, were developed through consultations with a wide range of stakeholder groups, and aim to facilitate consumer understanding of the food’s contribution to the energy and nutrient contents of a diet [4]. The proposals developed so far largely differ in many characteristics, such as being nutrient-specific or summary-indicator FOPL, and whether they are interpretative or informative [5].

Despite the large differences among the various FOPL proposals, they all have a double purpose. Indeed, on one hand, FOPL aims to help people make informed and possibly healthier food choices more easily [6,7,8]. On the other hand, FOPL aims to encourage food companies to formulate and reformulate their food products by improving their overall nutritional quality to achieve a more favorable label. Reformulation can contribute to improving the nutritional quality of diets at the population level, especially when the nutritional profiles of staple foods are improved, mainly in terms of reducing critical nutrients (e.g., salt, sugar, and saturated fat) or increasing nutrients that are consumed in lower amounts compared to dietary recommendations (e.g., fiber), thus representing an important public health tool [9,10,11]. 

Among the food groups that deserve particular attention for reformulation, cereal foods have been the focus of specific nutritional quality improvements in recent years by virtue of their nutritional characteristics and generally high levels of consumption in the population. In particular, bread is a staple food in many countries, including Italy, with a mean consumption of about 91 g per day, which is lower than the levels reported in the Italian dietary guidelines (IDGs), suggesting a daily consumption of 2.5–4.5 portions of 50 g each per day (corresponding to 125 to 225 g per day) depending on energy intake [12]. Despite this lower consumption, bread is the main source of salt and, consequently, sodium in the population; intake would increase if bread was consumed in line with the IDGs [13]. Therefore, in addition to finding methods to help people choose foods with the lowest salt content, it is important to find strategies to reduce the salt content in bread, such as through salt reduction or other methods [14,15]. In this scenario, it is noteworthy that Italy mainly consumes artisanal or homemade bread [15,16]. However, based on Regulation (UE) n. 1169/2011, nutritional declarations on food labeling are only mandatory for packaged foods. Moreover, the consumption of packaged bread is increasing due to its ease of use, longer shelf life, and convenience; thus, the reformulation of this food category is crucial to lowering salt intake in the population, which is much higher than the suggested dietary target of 5 g per day [17]. 

Based on these premises, the present study aimed to investigate whether FOPL (i.e., Nutri-Score and NutrInform battery) improves when food products are reformulated and whether these types of labeling serve as effective tools to encourage food reformulation by food companies. To this end, salt reduction in packaged breads was used as a case study as individuals’ salt intake remains much higher than dietary recommendations [16,17,18], and bread is the main source of salt intake in the Italian population [19].

## 2. Materials and Methods

### 2.1. Product Selection and Data Collection

Information about bread products to be used as a case study was collected from the major retailers present in the Italian market, as previously described by Angelino et al. [20]. The search was conducted in September 2023 and last updated in March 2024, and cross-sectional data were collected to represent a single point in time.

The information useful for calculating FOPL was retrieved from food labeling. In detail, the amounts of fruits, vegetables, legumes, and nuts were retrieved from the list of ingredients, whereas energy (kcal and kJ/100 g), sugar (g/100 g), total fat (g/100 g), saturated fat (g/100 g), and salt (g/100 g) were retrieved from the nutritional declaration. For a complete characterization of the products, additional information was collected, including the carbohydrate content (g/100 g), protein content (g/100 g), the presence and type of nutrition claims according to Reg. (UE) n. 1924/2006 [21], the presence of gluten-free declarations, and the presence of organic declarations. Based on descriptive names, the products in each category were sub-grouped into the following types: (A) loaves, (B) rolls, and (C) sliced bread.

The precision of the extracted data was double-checked by two independent researchers, and disagreements were resolved through secondary extractions with the help of a third researcher. 

### 2.2. FOPL Calculation

Among the different types of FOPL proposed so far, the Nutri-Score (NS) and NutrInform battery (NIB) were calculated in the present study. The NS is a graphic FOPL that categorizes foods into 5 classes (expressed by a color ranging from dark green to dark orange and a letter ranging from A to E) [22]. 

The NS was calculated for each item, before and after reformulations, in accordance with the latest document. Briefly, the score was calculated using the data from the nutritional declaration per 100 g of product and the ingredient list, attributing points to the contents of “unfavorable” elements (i.e., energy in kJ, sugar, salt, and saturated fatty acids) and “favorable” elements (i.e., fiber, fruits, vegetables, legumes, nuts, and eventually protein). The final score for each item was calculated by subtracting the total number of favorable points from the total number of unfavorable points. 

The NIB appears as five boxes indicating the quantities of energy (in kJ and kcal), fat, saturated fat, sugar, and salt (in grams) of a single portion (i.e., 50 g of bread). Inside the “battery” symbol, the percentages of these components contained in each single portion are shown in relation to the suggested reference intake for adults as defined by the Reg. (UE) n. 1169/2011 [23]. The NIB for salt was instead calculated as a box indicating the content of salt per portion, and the battery was calculated as the percentage of salt contained in 50 g of bread in relation to the suggested daily quantity of salt of 6 g/day [4].

### 2.3. Selection of Reformulation Strategies

To examine potential changes in FOPL following reformulation, three different hypothetical strategies were selected: (i) a 25% salt decrease compared to the current median salt content in bread, which represents the reduction needed to bear the nutrition claim “reduced in salt/sodium” in accordance with Reg. (CE) n. 1924/2006 [21]; (ii) a reduction of up to 330 mg/100 g of sodium (0.825 g/100 g of salt) corresponding to the benchmark set by the World Health Organization (WHO) [24]; and (iii) the minimum salt reduction needed to improve the NS by one grade (e.g., from D to C).

### 2.4. Statistical Analysis

Statistical analyses were carried out using IBM SPSS Statistics for Macintosh, Version 29.0. Armonk, NY, USA: IBM Corp, setting the significance level at *p* < 0.05. The variables are expressed as the median and interquartile range since the normal distribution of data was rejected using the Kolmogorov–Smirnov test. Differences in the salt content per 100 g among product types and NS categories were assessed using the Kruskal–Wallis non-parametric one-way ANOVA for independent samples with multiple pairwise comparisons, whereas differences between GF declaration and nutrition claim categories were analyzed using the Mann–Whitney non-parametric test for two independent samples.

## 3. Results

### 3.1. The Characteristics of the Selected Products

A total of 527 items of packaged bread currently sold in Italy were selected, of which 184 were products of the “loaf” type (35%), 112 were “rolls” (21%), and 231 were “sliced bread” products (44%). Nutrition claims were observed in 194 products (36.8%). In particular, the most used nutritional claim in this product category was the one on fiber content (n = 160), followed by claims on fats (n = 32) and salt (n = 15). A total of 62 items (12%) reported an organic declaration, while 52 (10%) were gluten-free products. Regarding the salt content, the median content was 1.3 (1.1–1.4) g/100 g with no statistically significant differences among types (i.e., “loaf”: 1.3 (1.0–1.5) g/100 g; “rolls”: 1.3 (1.2–1.5) g/100 g; and “sliced bread”: 1.3 (1.2–1.4) g/100 g). The amount of 1.3 g/100 g of salt is equivalent to 0.520 g/100 g of Na, which is more than 50% higher than the benchmark value of 0.330 g/100 g of Na (i.e., 0.825 g/100 g of salt) set by the WHO for this category of products. In detail, only 44 out of 527 products were below this value (8%), while the remaining ~92% were above and had an average content of 1.3 g salt/100 g (Table 1).

### 3.2. FOPL in Current Products

Regarding FOPL, the salt content and thus the related battery in the NIB was, on average, 11% of the reference intake in the Reg. (UE) n.1169/2011 (i.e., 6 g), with 82% of the products having a content between 8 and 13%. However, a large variability was observed, with values ranging from 0% to 23% of the reference intake. Large variability was also observed for the NS, with most of the products scoring “C” (n = 278; 52.8%) and “D” (n = 124; 23.5%), followed by “A” (n = 71; 13.5%) and “B” (n = 48; 9.1%), while only six products (1.1%) scored “E”.

**Figure 1:**
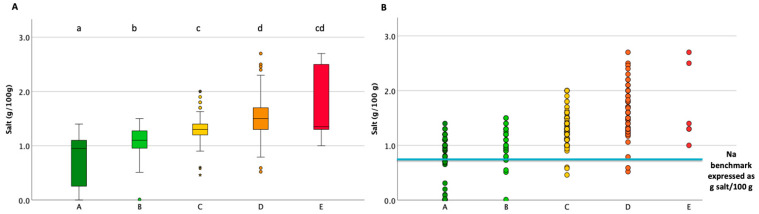
The current Nutri-Scores of all packaged bread on the market (**A**), stratified for having a sodium content below or above the benchmark (**B**) of 330 mg of sodium (corresponding to 0.825 g salt/100 g). Different letters or asterisks indicate significant differences among types (the Kruskal–Wallis test was used for independent samples with multiple pairwise comparisons, and the Mann–Whitney non-parametric test was used for two independent samples) (*p* < 0.05).

In general, the products scoring “A” contained a statistically significant lower salt content than the other categories, and the salt content increased significantly to the “D” class, as shown in Figure 1A. Items above the WHO benchmark for sodium were found in all five NS categories, while no products below the benchmark scored “E” (Figure 1B).

### 3.3. Impact of Reformulation on FOPL

#### 3.3.1. Scenario of 25% Salt Reduction

The first hypothetical scenario involved a 25% reduction in salt compared to the median content in packaged bread on the market. Since the median salt content was 1.26 g/100 g (corresponding to 500 mg sodium/100 g), it was hypothesized to reduce this value to the target of 0.95 g/100 g (380 mg sodium/100 g). The distance from this value was 0.41 g on average, and specifically, it was 0.49 g for “loaf”, 0.42 g for “rolls”, and 0.34 g for “sliced bread”.

**Figure 2:**
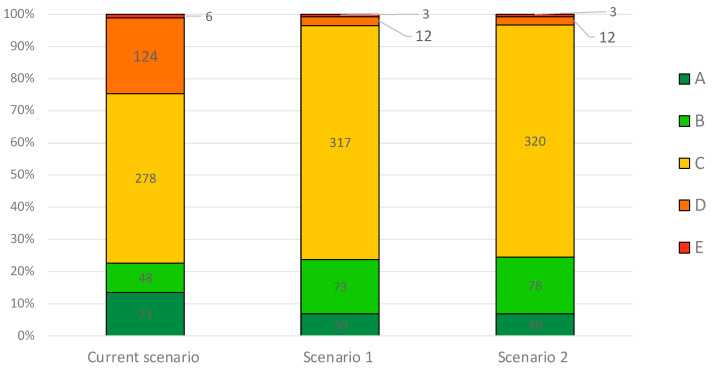
Nutri-Scores in the current samples (n = 527) and after reformulation via a 25% salt reduction (Scenario 1, n = 435 *°) up to the WHO benchmark (Scenario 2, n = 443 *^§^). * The results after removing products with a Nutri-Score of A. ° The results after removing products with a salt content below the median content in bread on the market. ^§^ The results after removing products with a sodium content already below the WHO global benchmark.

There were 471 products with a salt content above the target, and the impact of reformulation was estimated in the 435 products that did not score “A” and therefore could not improve the NSs (122 products of the “loaf” type, 106 “rolls”, and 207 “sliced” bread products). Of these 435 products, 196 (45.1%) had improved NSs (Figure 2).

As regards the salt battery of the NIB, 431 (126 products of the “loaf” type, 102 “rolls”, and 203 “sliced bread” products) out of the 471 products reduced the percentage of reference intake per portion to 8% thanks to the 25% salt reduction (Table 2). Conversely, in the remaining food products, the NIB was barely affected by reformulation as minimal salt reduction is needed to reach the target value of 0.95 g/100 g (i.e., between 0.02 and 0.05 g/100 g).

#### 3.3.2. The Scenario of Reduction up to the WHO Benchmark

In the second hypothetical scenario, the salt reduction was postulated to reach the benchmark of 0.330 g/100 g of sodium set by the WHO (i.e., 0.825 g/100 g of salt). Thus, out of the 483 products that had a salt content higher than the benchmark, reduction was hypothesized with the aim of reaching the value of 0.825 g/100 g.

On average, the salt reduction to be applied was 0.52 g, ranging from 0.075 g to 1.875 g, and the impact of this reformulation scenario on the NS and NIB was calculated for 443 products after excluding the other 40 items already scoring “A” in the NS.

A total of 196 products (44%) had improved NSs. Among the remaining 247 products that did not improve their NSs, 34 had a salt content very close to the benchmark (i.e., delta lower than 0.2 g); therefore, the reformulation scenario was not able to minimally shift the salt score, which has a sensitivity of 0.2 g/100 g. Overall, compared to the current scenario, this scenario would lead to an increase in the number of products classified as A, B, and C and a decrease in those classified as D and E (Figure 2).

As regards the salt battery of the NIB, all 483 products with a salt content above the benchmark (150 products of the “loaf” type, 109 “rolls”, and 224 “sliced bread” products) had a reduced percentage of the reference intake following reformulation, bringing it to 7% (Table 2).

#### 3.3.3. The Scenario of Minimal Reduction to Improve the Nutri-Score

**Figure 3:**
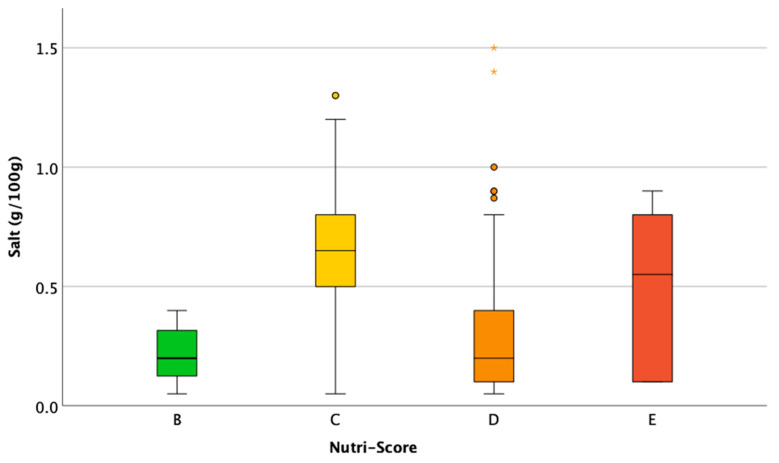
The minimal amount of salt (g/100 g) to remove to improve the Nutri-Score. Dots and asterisks represent outliers and extreme outliers, respectively.

The third and final hypothetical scenario involved a minimum reduction in the quantity of salt that is sufficient to improve the NS of the product. This theoretical reformulation scenario was applied to 445 (129 loaves, 106 rolls, and 210 sliced bread products) out of 527 products. A total of 82 products were excluded from the reformulation since they had an NS of A (71 products), a salt content ≤ 0.2 g/100 g (1 product), or an insufficient quantity of salt to make the necessary reduction to improve the score (10 products). All 445 products had improved NSs by applying an average reduction of 0.49 g (min 0.05 g; max 1.5 g) with a median of 0.50 g (0.20–0.70 g). Of these, 83 products (19%) had improved NSs with a salt reduction intervention of less than 0.20 g (between 0.05 g and 0.18 g). The amount necessary to remove to improve the NS by one grade widely varied depending on the grade, ranging from an average of 0.24 g (median 0.20 g (0.13–0.32 g)) to improve the score from B to A to 0.62 g of salt (median of 0.65 g (0.50–0.80 g)) to improve it from C to B (Figure 3). The salt battery of the NIB was reduced in 437 out of the 445 reformulated products; of the other 8, reformulation did not impact the percentage of reference intake for salt because the salt reduction was minimal, i.e., 0.05 g.

## 4. Discussion

FOPL should aim to encourage the reformulation of food products with the intention to improve their nutritional quality, thus representing an important tool for public health.

The impact of FOPL on food choices and eating behavior has been investigated in the literature with contrasting results [25,26,27]. In general, FOPL schemes that use colors seem to be preferred and more effective than monochrome ones, although there are differences based on country and type of consumer [5]. While the impact of FOPL on food preference and food choices has been the object of several studies, the relation between food (re)formulation and FOPL has been scarcely investigated.

As demonstrated in the literature, the introduction of FOPL in different countries has stimulated the reformulation of numerous food categories. For instance, van der Bend et al. [28] observed a significant reduction in the contents of energy, sodium, trans-fat, SFA, and added sugar in several product categories with the Dutch Choices Logo analyzed between 2006 and 2016, suggesting the importance of FOPL in product reformulation. Similarly, Vermote and colleagues [29] noticed a significant reformulation of cereals between 2017 and 2018 in anticipation of the Nutri-Score’s implementation, with reductions in the total sugar and sodium contents and increases in fiber.

As FOPL is based on the nutritional characteristics of food products, it represents an effective strategy to reduce the consumption of critical nutrients, such as salt and sugar, and to reduce the burden of non-communicable diseases associated with their excessive intake [30,31]. This was recently demonstrated by Sarda et al. [32], who observed an improvement in diet quality, i.e., significant reductions in energy, SFA, sugar, and salt intake and an increase in fiber after theoretical reformulation using real food market data in France. Similarly, two studies found a significant reduction in the intake of several critical nutrients following theoretical food reformulation in two Australian national nutrition surveys on the general population and in children [33,34].

In the present study, we investigated the impact of different hypothetical scenarios involving the reformulation of two different types of FOPL (i.e., the Nutri-Score, as an example of synthetic and interpretative FOPL, and NutrInform battery, as an example of informative and non-interpretative FOPL). In detail, we simulated reformulation through salt reduction in packaged bread, since this is a staple food representing the main contributor to salt intake in the Italian population [35].

Among the three theoretical scenarios, the first one involved simulating salt reduction with the aim of reaching the 25% salt reduction needed to bear the comparative nutrition claim “reduced in salt” in accordance with Regulation (CE) n. 1924/2006, which could encourage reformulation by food companies to make a food product easier to distinguish when compared to a range of foods of the same category [21]. This reformulation scenario would translate to an average reduction in salt content of 0.3 g/100 g, thus making it possible to reduce salt intake by ~0.5 g per day for 2000 kcal when bread is consumed in line with Italian dietary guidelines [12]. Therefore, this reformulation scenario can represent a major contributor to achieving the reduction in salt intake to 5 g/day as recommended at the national and international levels [17]. However, reformulation shall consider the technological aspects (e.g., from production to the preservation/shelf life of the product), which may be particularly important for specific foods (e.g., gluten-free products). Moreover, it should be considered that reformulation may impact food quality and thus the consumer’s food choices. In this regard, it cannot be excluded that reformulation in the salt content of bread could have a negative impact on its sensory properties, thus affecting the consumer’s food choices. There is little evidence within this context, but a previous study seemed to document that up to a 40% reduction in salt content does not impact the consumer’s acceptability [36] despite other studies suggesting a small stepwise reduction to maintain consumer acceptance [37]. In the present study, reduction up to the target of 0.95 g/100 g improved the Nutri-Score in less than half of the products, while the majority had improved NIB. 

Similar results were observed in the second scenario when the target value was set at 0.825 g/100 g, corresponding to the benchmark of 330 mg sodium/100 g set by the WHO [24]. Only a small portion of products included in the present study had a sodium content lower than the benchmark, which is in accordance with a previous study reporting that most bread and bread alternatives on the Italian market exceed this value [38]. When the theoretical reformulation of the products was simulated, we found that about half of the products had improved Nutri-Scores, and all had improved NIB, which could encourage healthier food choices by consumers and lead to a significant reduction in salt intake (by an average of 0.4 g/day considering the current level of bread consumption in Italy). However, it is noteworthy that the mean reduction of ~0.5 g/100 g needed to reach the benchmark for all items corresponds to a reduction of about 40% compared to the current content. Thus, it would be important to understand the impact of this reformulation scenario on consumer acceptability in which maintenance may represent a major challenge [36].

The last scenario was hypothesized to simulate possible reformulation aimed only at improving FOPL. In this scenario, we found that minimal reformulation in terms of salt reduction (i.e., <0.2 g/100 g) can be sufficient to improve the Nutri-Score in some products. Although this reformulation scenario is likely also feasible from technological and sensory points of view, it barely improves the nutritional quality since a reduction of 0.2 g/day would correspond to a reduction of 0.18 g/day considering the current bread consumption rates in Italy. Moreover, this reduction may even mislead consumers to believe that they are consuming a product with increased nutritional quality [39,40].

A thorough comparison of the results from the present study with previous findings is not simple since, to the best of our knowledge, this is the first study investigating the impact of salt reduction on two different types of FOPL.

In a previous study, ter Borg and colleagues [41] investigated whether the Nutri-Score could be used as an incentive for reformulation by considering whether a one-point decrease in the Nutri-Score subscore (e.g., 90 mg/100 g of sodium, 1 g/100 g of SFA, or 4.5 g/100 g of sugar) could result in a more favorable Nutri-Score. The authors found that a reduction in sodium, SFA, or sugars resulted in a more favorable Nutri-Score in different food groups. However, the Nutri-Scores improved following a 90 mg/100 g decrease in the sodium content for only 12% of “meat, poultry, and meat preparation” products and in 7% of “cheeses”. Thus, in line with the present study in which less than half of the products would benefit from a reduction in the salt content up to the median content and to the benchmark set by the WHO, the results seem to suggest that reformulation might not affect the FOPL in many cases, which could discourage the reformulation of these products.

The present work has some limitations worth noting. First, we simulated reformulation by only reducing the salt content, but salt reduction is often more complex than simple salt removal, with salt in bread, among other ingredients, being involved in controlling the growth of yeast and fermentation and in making gluten more stable. For this reason, reformulation often requires rebalancing the recipe and can cause various technical hurdles for food manufacturers [9,42,43]. This rebalancing may also have an impact on other critical nutrients, some of which are also included in FOPL (e.g., sugar, fiber), and may further change following reformulation. Moreover, as it was hypothetical, reformulation was simulated without considering the formulation and the impact on the technological and sensory properties of the foods, which should be carefully considered during product reformulation to limit the reduction in acceptability by consumers [43,44]. Finally, we only focused on two different types of FOPL despite others having been proposed so far. However, these two types of FOPL were chosen by virtue of the different approaches used for their development (e.g., interpretative vs. not interpretative; nutrient-based vs. synthetic).

## 5. Conclusions

In conclusion, the present work can contribute to the current debate about the impact of FOPL in promoting food quality and choices. Reformulation represents an effective strategy to improve food quality and, in turn, diet quality among the population and should thus be promoted in conjunction with other policies. However, FOPL is not always effective in capturing food reformulation, possibly discouraging the efforts of food companies to improve the nutritional quality of foods which should instead be stimulated to help people make healthy food choices. We found that nutrient-specific types of FOPL seem to be more effective than synthetic ones in highlighting food reformulation, especially if this is focused only on one component such as salt. This is because synthetic FOPL considers multiple components and thus may not change despite reformulation aimed at increasing or reducing one of these components. Future studies on other food categories and other critical nutrients should be performed to better elucidate the impact of reformulation on different types of FOPL. These steps will be pivotal for better elucidating whether and which types of FOPL may be useful tools for encouraging food companies to reformulate food products in a real-life situation.

## Figures and Tables

**Table 1:** Salt and the corresponding sodium contents in the retrieved samples.

	Salt (g/100 g)	Sodium (mg/100 g)
All breads (n = 527)	1.3 (1.1–1.4)	520 (440–560)
*Loaves* (n = 184)	1.3 (1.0–1.5) ^a^	520 (400–600) ^a^
*Rolls* (n = 112)	1.3 (1.2–1.5) ^a^	520 (480–600) ^a^
*Sliced bread* (n = 231)	1.3 (1.2–1.4) ^a^	520 (480–560) ^a^
Gluten-free (n = 52)	1.3 (1.0–1.5)	520 (440–600)
Not gluten-free (n = 475)	1.3 (1.1–1.4)	520 (440–560)
Organic (n = 62)	1.2 (0.9–1.3)	480 (360–520)
Conventional (n = 465)	1.3 (1.1–1.5) *	520 (440–600) *
With NCs (n = 194)	1.2 (1.0–1.3)	480 (400–520)
Without NCs (n = 333)	1.3 (1.2–1.5) *	520 (480–600) *
With NCs on salt (n = 15)	0.01 (0.01–0.02)	4 (4–8)
Without NCs on salt (n = 512)	1.3 (1.1–1.4) *	520 (440–560) *
Na below benchmark (n = 44)	0.5 (0.0–0.7)	200 (0–280)
Na above benchmark (n = 483)	1.3 (1.2–1.5) *	520 (480–600) *

Values are expressed as median (25–75th percentile). Different letters or asterisks indicate significant differences among types (Kruskal–Wallis test was conducted for independent samples with multiple pairwise comparisons, and Mann–Whitney non-parametric test was conducted for two independent samples) (*p* < 0.05). Legend: NCs, nutrition claims.

**Table 2:** The NutrInform battery (NIB) for salt in the current samples and after reformulation via a 25% salt reduction (Scenario 1) up to the WHO benchmark (Scenario 2) or minimal reformulation to improve the Nutri-Score (Scenario 3).

	Current Scenario	Scenario 1 °	Scenario 2 ^§^	Scenario 3 ^#^
Number of items improving salt NIB	-	431/471	483/483	437/445
Salt content (g/portion)	0.65 (0.55–0.70)	0.48 (0.48–0.48)	0.41 (0.41–0.41)	0.40 (0.30–0.60)
Sodium content (mg/portion)	260 (220–280)	192 (192–192)	164 (164–164)	160 (120–240)
% of salt compared to reference intake	10.8 (9.2–11.7)	7.9 (7.9–7.9)	6.9 (6.9–6.9)	6.7 (5.0–10.0)

° The results after removing products with a salt content lower than the median (n = 471). ^§^ The results after removing products with a sodium content already below the WHO global benchmark (n = 483). ^#^ The results after removing products with a Nutri-Score of A, a salt content ≤ 0.2 g/100 g, or an insufficient salt quantity to make the necessary reduction to improve the score. The data are expressed as the median (25–75° percentile) in g of salt per portion and in the percentage of salt compared to the daily reference intake as defined in the Reg. (UE) n. 1169/2011 (i.e., 6 g).

## Data Availability

The original contributions presented in the study are included in the article, further inquiries can be directed to the corresponding author.
